# Increased Incidence of Colon Tumors in AOM-Treated *Apc*^1638N/+^ Mice Reveals Higher Frequency of Tumor Associated Neutrophils in Colon Than Small Intestine

**DOI:** 10.3389/fonc.2019.01001

**Published:** 2019-10-02

**Authors:** Rebecca Metzger, Mahulena Maruskova, Sabrina Krebs, Klaus-Peter Janssen, Anne B. Krug

**Affiliations:** ^1^Biomedical Center, Institute for Immunology, Ludwig-Maximilians-University Munich, Munich, Germany; ^2^Department of Surgery, Klinikum rechts der Isar, Technische Universität München, Munich, Germany

**Keywords:** colorectal cancer, mouse model, adenomatous polyposis coli, azoxymethane, tumor immunology and microenvironment, tumor-associated neutrophils, tumor-associated macrophages, dendritic cells

## Abstract

Colorectal cancer (CRC) is one of the most common cancers and a major cause of mortality. Mice with truncating *Apc* germline mutations have been used as a standard model of CRC, but most of the *Apc*-mutated lines develop multiple tumors in the proximal small intestine and rarely in the colon precluding detailed analysis of colon tumor microenvironment. Our aim was to develop a model with higher resemblance to human CRC and to characterize tumor infiltrating immune cells in spontaneously developing colon tumors compared to small intestinal tumors. Therefore, the *Apc*^1638N/+^ line was treated repeatedly with azoxymethane (AOM) and 90% colon tumor incidence and 4 to 5 colon tumors per mouse were achieved. Of note, AOM treatment specifically increased the tumor burden in the colon, but not in the small intestine. Histological grading and WNT-signaling activity did not differ significantly between small intestinal and colon tumors with some lesions progressing to invasive adenocarcinoma in both locations. However, characterization of the intratumoral myeloid cell compartment revealed a massive infiltration of colon tumors with neutrophils − 6-fold higher than in small intestinal tumors. Moreover, CCL17-expressing macrophages and dendritic cells accumulated in the tumors indicating the establishment of a tumor-promoting immunosuppressive environment. Thus, *Apc*^1638N/+^ mice treated with AOM are a suitable and straightforward model to study the influence of immune cells and chemokines on colon carcinogenesis.

## Introduction

Colorectal cancer (CRC) is one of the most prevalent cancers worldwide, and one of the leading causes of cancer-related morbidity and mortality, especially in countries with “Western” life style. Aberrant WNT signaling plays an important role in initiation of human colorectal carcinogenesis. Loss of the adenomatous polyposis coli (*APC*) tumor suppressor is not only the cause of familial adenomatous polyposis, but also 80–90% of sporadic CRC harbor loss of function mutations—mostly truncating nonsense mutations—in the *APC* gene (1). The second *APC* allele is inactivated by promoter methylation, chromosomal loss, or additional mutations leading to biallelic loss (loss of heterozygosity, LOH) or inactivation of *APC* (2, 3). As a result, β-catenin is not degraded, accumulates and translocates to the nucleus where it acts as a transcriptional coactivator inducing the expression of WNT target genes including c-Myc, Cyclin D1, and osteopontin which promote proliferation and ultimately tumor formation (2, 4). Additional mutations, e.g., in *KRAS, PTEN, PIK3CA, TGFBR1, TGFBR2, SMAD2, SMAD4*, and *TP53* are found in *APC*-mutated sporadic CRC, which promote tumor progression. In contrast, CRC lacking *APC* mutations are frequently associated with mutations in mismatch repair genes (2).

Mice heterozygous for truncating germline mutations of *Apc*, such as the *Apc*^Min/+^ line on C57BL/6 background (5) have been used for decades as a preclinical model. A major disadvantage of the frequently used *Apc*^Min/+^ model is however that the mice quickly develop multiple adenomas in the small intestine (SI), and only few polyps in the colon, which leads to a life span of <6 months on the C57BL/6 background (2). Further, progression of these lesions to invasive adenocarcinoma is very rare (2).

*Apc*^1638N/+^ mice were generated by inserting a neomycin cassette in antisense orientation into exon 15, resulting in chain termination at codon 1638 and production of an unstable protein. These mice develop intestinal adenomas and adenocarcinomas, which was attributed to somatic loss of the wildtype *Apc* allele (6) or rather *Apc* mutations as described in a more recent publication (7). In comparison to *Apc*^Min/+^ mice the *Apc*^1638N/+^ mice develop less tumors with longer latency and show progression to invasive adenocarcinomas, as well as splenomegaly and desmoid formation, thus more closely resembling human CRC. Although colon tumors develop in *Apc*^1638N/+^ mice, their incidence and number is low and their formation takes 10–12 months (2, 6, 8). Treating *Apc*^1638N/+^ mice with “Western style” diet (9) or crossing them with other genetically engineered mutant or knockout mice (6, 10–17) promotes multiplicity and sometimes progression of tumors in SI and/or colon. Similarly, conditional knockout of *Apc* in colonic epithelial cells leads to selective colon tumor formation (18, 19).

However, mice carrying several mutant or transgenic alleles are cumbersome to work with for mechanistic studies, which require crossing these models with additional knockout or transgenic mice. Moreover, when using a Cre-mediated knockout of *Apc* in colonic epithelial cells for tumor formation, alternative recombinase systems need to be used for conditional gene knockouts in other cell types.

Induction of colitis by administration of dextran sodium sulfate (DSS) greatly accelerates adenoma and adenocarcinoma formation in the colon of *Apc*^Min/+^ mice (20, 21), but this model is not suitable to study tumorigenesis in the absence of overt inflammation, which would better mimick human CRC pathogenesis. Administration of azoxymethan (AOM), an alkylating agent that produces free radicals, to C57BL/6 mice leads to low incidence tumor formation in the colon by causing mutations in β-catenin (22). AOM treatment increased incidence and numbers of colon adenomas and adenocarcinomas in adult *Apc*^Min/+^ mice (23) and in young or neonatal *Apc*^Min/+^ mice, but these mice still had a predominance of small intestinal tumors and a short life span (24–26). The histological features of tumors from these mice have been described but a detailed characterization e.g., regarding immune cell infiltration is lacking.

Studies performed in the *Apc*^Min/+^ model indicate that tumors are controlled by the adaptive immune system (27, 28). However, regulatory T cells and myeloid cells, such as tumor associated neutrophilic granulocytes (TAN) and monocytic cells (functionally described as myeloid derived suppressor cells, MDSC) as well as tumor associated macrophages (TAM) together shape the tumor microenvironment to promote tumor growth and to limit the anti-tumor immune response (29–33). A subpopulation of TAMs isolated from subcutaneous tumor models was shown to secrete CCL17 as a hallmark of M2-like macrophage polarization (34). Unfortunately, the low incidence and number of spontaneous colon tumors in *Apc*^Min/+^ and *Apc*^1638N/+^ mice precludes a detailed characterization of immune cell infiltrates including TAMs and TANs in spontaneously developing colon tumors. We therefore established an accelerated *Apc*^1638N/+^ model which is suitable for further investigations of the tumor microenvironment and anti-tumor immune responses in spontaneously forming colon tumors. In this study we show that repeated administration of AOM to *Apc*^1638N/+^ mice leads to a higher incidence and an increased number of tumors in the colon and shortens experimental time to 6–7 months.

## Results

### Repeated Injection of AOM Leads to Higher Incidence and Multiplicity of Colon Tumors and Decreased Survival in *Apc*^1638N/+^ Mice

**Figure 1:**
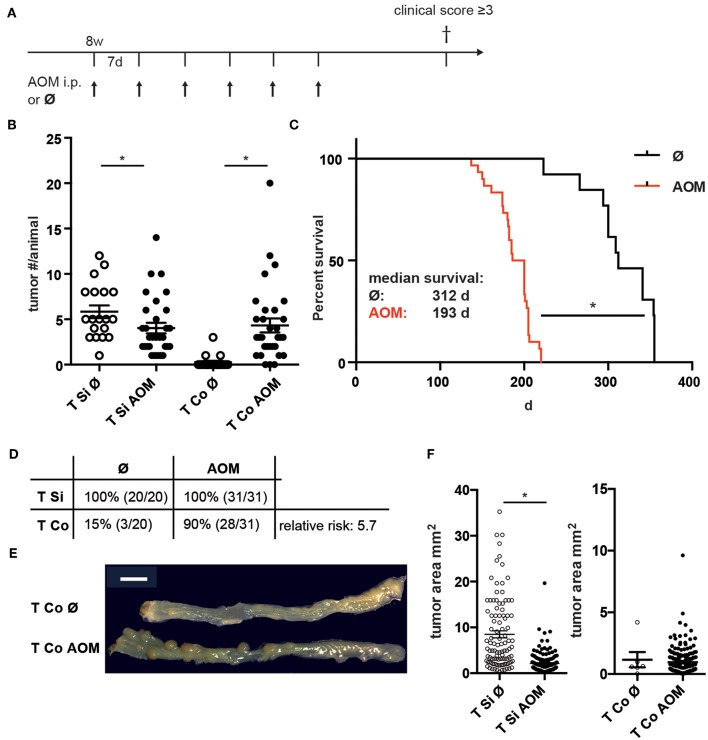
Increased incidence and number of colon tumors in AOM-treated APC^1638N/+^ mice. **(A)** Experimental scheme. *Apc*^1638N/+^ mice received weekly i.p. injections of AOM from age 8 to 14 weeks and were sacrificed when reaching criteria for euthanasia. **(B)** Number of tumors in small intestine (Si) and colon (Co) in untreated (*n* = 19) and AOM-treated (*n* = 31) mice (Mann–Whitney test). **(C)** Kaplan-Meier survival analysis (log-rank test). **(D)** Tumor incidence in Si and Co (Chi-square analysis). **(E)** Representative macroscopic image of colon tumors in untreated and AOM-treated *Apc*^1638N/+^ mice. Scale bar: 1 cm. **(F)** Tumor sizes of Si and Co tumors (Si untreated: *n* = 103, Si AOM: *n* = 150, Co untreated: *n* = 5, Co AOM: *n* = 189, *p < 0.0001, Mann–Whitney test). *p < 0.05.

To generate more colon tumors in a shorter experimental time, adult C57BL/6 mice carrying one CCL17-eGFP knock-in allele as a reporter for CCL17 expression received weekly AOM injections for 6 weeks, and were followed by clinical assessment until anemia was clinically apparent or other criteria for euthanasia were reached (Figure 1A). While the number of colon tumors per mouse (Figure 1B) was significantly higher in AOM treated vs. untreated *Apc*^1638N/+^ mice (median 3 vs. 0; mean ± SEM 4.3 ± 0.8 vs. 0.3 ± 0.2), the number of macroscopically visible tumors in the SI was slightly reduced in AOM treated vs. untreated *Apc*^1638N/+^ mice (median 3 vs. 5; mean ± SEM 4.0 ± 0.6 vs. 5.8 ± 0.7). Survival time was significantly reduced in AOM treated *Apc*^1638N/+^ mice with a median survival time of 193 days compared to 312 days in untreated mice (Figure 1C). The cumulative incidence of colon tumor development increased 6-fold from 15 to 90% after the administration of AOM, with a 5.7-fold higher relative risk for colon tumor development with AOM treatment (*p* < 0.0001, Chi-square test, Figure 1D). Colon tumors were macroscopically similar and were localized in the distal half of the colon in AOM treated and untreated *Apc*^1638N/+^ mice (Figure 1E). Interestingly, tumors in the SI were found to be significantly smaller in the AOM-treated mice (median 1.3 vs. 5.3 mm^2^; Figure 1F).

Thus, administration of AOM to adult C57BL/6 *Apc*^1638N/+^ mice specifically accelerates tumor development in the colon, but not in the small intestine leading to a higher incidence and multiplicity of colon tumors.

### AOM-Treated *Apc*^1638N/+^ Mice Develop Highly Proliferative Colon Tumors With Active Wnt Signaling and Aberrant Accumulation of β-Catenin

**Figure 2:**
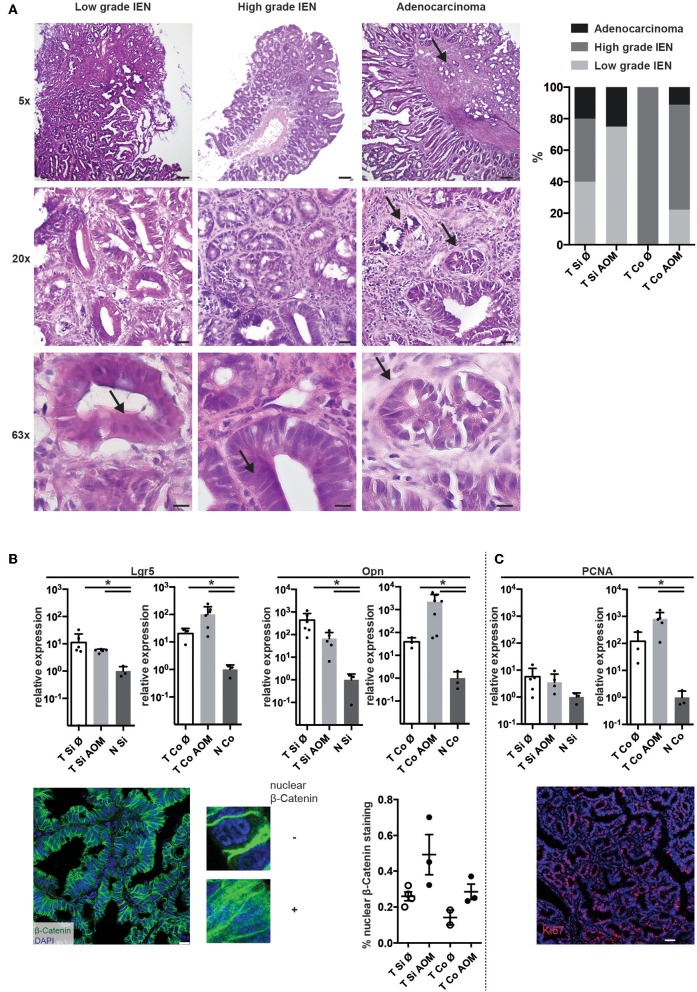
AOM-treatment leads to the development of high-grade colonic lesions with active Wnt-signaling and proliferation. **(A)** Exemplary images of H&E stained cryosections of tumors isolated from AOM-treated and non-treated *Apc*^1638N/+^ mice (taken with 5×, 20×, and 63× objectives). Scale bars: 100 μm (5×), 25 μm (20×), 10 μm (63×). Arrows indicate features of cell dedifferentiation and dysplasia such as pseudo-stratification, increased nucleus/cytoplasm ratios and abnormal nuclei positioning as well as invasion of the muscularis mucosae in adenocarcinomas. Proportions of low grade IEN, high grade IEN and adenocarcinoma are shown in the graph (Si untreated, *n* = 5; Si AOM *n* = 4; Co untreated, *n* = 1; Co AOM, *n* = 9). **(B)** Upper panel: relative mRNA expression of WNT target genes *Lgr5* and *Opn* in tumors and normal intestinal tissue from tumor-bearing mice was measured by qRT-PCR (fold-change compared to normal tissue average, log(10) scale, mean + SEM, *n* = 3–6). Lower panel, left: representative image of immuno-fluorescence staining for β-Catenin in cryosections of a colon tumor. Scale bar: 50 μm. Zoomed images show nuclear and extranuclear localization of β-Catenin. Green: β-Catenin, blue: DAPI, 63× magnification. Right: quantification of nuclear β-Catenin staining of total β-Catenin staining in one field of view per mouse. Symbols indicate tumors from individual mice; mean + SEM (*n* = 2–3). **(C)** Upper panel: relative mRNA expression of *Pcna* in tumors and normal intestinal tissue from tumor-bearing mice was measured by qRT-PCR (fold-change compared to normal tissue average, log(10) scale, mean + SEM, *n* = 3–6, unpaired, two-tailed *t*-test). Lower panel: representative Ki67 staining of intestinal tumor tissue of an *Apc*^1638N/+^ mouse (20×, red: Ki67, blue: DAPI, Scale bar: 50 μm). *p < 0.05.

Histopathological examination of tissue sections from colon and SI tumors at the time points of sacrifice revealed low grade and high grade intraepithelial neoplasia (IEN) with distorted crypt architecture, high nuclear to cytoplasmic ratio, and elongated stratified hyperchromatic nuclei, which in high grade IEN reached the luminal side. In some tumors invasion of the muscularis mucosae with stromal and inflammatory reactions was observed indicating progression to adenocarcinoma (Figure 2A). Low grade IEN, high grade IEN and occasionally adenocarcinomas were detected in intestinal tumors of both AOM-treated and untreated mice. High grade IEN was observed in colon tumors from AOM treated mice (6/9), but not in SI tumors of AOM treated mice (0/4) (Figure 2A).

Expression of canonical Wnt target genes, such as the stemness marker *Leucine-rich repeat-containing G-protein coupled receptor 5* (*Lgr5*) (35) and *osteopontin* (*Opn, Spp1*) (4) on mRNA level was significantly higher in SI and colon tumors in comparison to normal intestinal tissue. A trend towards higher expression of *Lgr5* and *Opn* in colon tumors of AOM treated *Apc*^1638N/+^ mice was observed (Figure 2B, upper panels). Strong β-catenin staining with cytoplasmic and nuclear localization [in contrast to membranous staining found in normal epithelium (4, 36)] was detected in all tumors with a trend toward a higher proportion of nuclear β-catenin staining in AOM-treated mice (Figure 2B, lower panels). Ki67 staining in tumor tissue sections confirmed the high percentage of proliferating cells in the tumors (representative result shown in Figure 2C, lower panel). Accordingly, expression of *Proliferating cell nuclear antigen* (*Pcna*) mRNA was significantly higher in colon tumors compared to normal colon tissue and slightly higher in colon tumors of AOM treated than untreated mice (Figure 2B). Thus, tumors developing in AOM-treated *Apc*^1638N/+^ mice are highly proliferative and show aberrant distribution of β-catenin and active WNT signaling.

### Comparison of Immune Cell Infiltrates in Small Intestinal and Colon Tumors Reveals Preferential Accumulation of Neutrophilic Granulocytes in Colon Tumors

**Figure 3:**
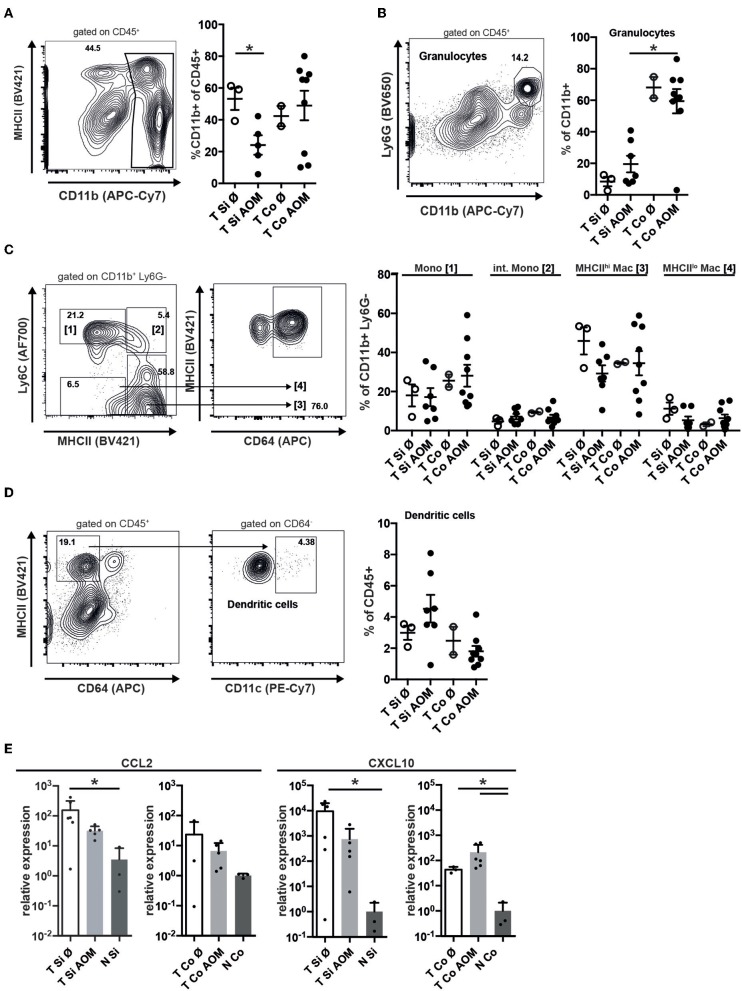
Accumulation of granulocytes in intestinal tumors correlates with chemokine expression and is enhanced in colon tumors. **(A)** Representative contour plot of intratumoral (AOM treated colon tumor) immune cells stained for MHCII and CD11b, showing the gating for CD11b^+^ cells. The percentages of CD11b^+^ cells of all CD45^+^ cells in Si and Co tumors of untreated and AOM treated *Apc*^1638N/+^ mice are shown in the graph. **(B)** Representative dot plot of intratumoral CD45^+^ immune cells stained for Ly6G and CD11b, showing the gating for CD11b^+^ Ly6G^+^ granulocytic cells. The percentages of CD11b^+^ Ly6G^+^ cells of all CD11b^+^ cells are shown in the graph. **(C)** Gating strategy for monocytes (Mono), intermediate monocytes (int. Mono), and MHCII^lo^/^hi^ macrophages within the intratumoral CD11b^+^ Ly6G^−^ population. The percentages of the indicated populations within the CD11b^+^ Ly6G^−^ cells are shown in the graph. **(D)** Gating strategy for intratumoral DCs and frequency of DCs within CD45^+^ cells. **(A–D)** Each symbol represents an individual mouse. Horizontal bars indicate mean, error bars indicate SEM, *n* = 2–9. **(E)** Relative mRNA expression of the chemokines CCL2 and CXCL10 in tumors and normal intestinal tissue from AOM-treated and untreated *Apc*^1638N/+^ mice measured by qRT-PCR [fold-change compared to normal tissue, log(10) scale]. Symbols indicate individual mice, horizontal bars: mean, error bars: SEM, *n* = 3–6, unpaired, two-tailed *t*-test. *p < 0.05.

Immune cell infiltrates in human colorectal cancer are an important prognostic factor. Tumors in SI and colon of both AOM treated and untreated mice were strongly infiltrated with CD45^+^ cells (Supplementary Figures 1, 2). These were localized below the neoplastic epithelium either distributed throughout the tumor or forming clusters (Supplementary Figure 2). In all tumors CD45^+^ cells contained a substantial proportion of T lymphocytes, including CD4^+^ and CD8^+^ T cells as well as CD4^+^ Foxp3^+^ regulatory T cells and a smaller more variable proportion of CD19^+^ B lymphocytes (Supplementary Figure 1). CD11b^+^ myeloid cells — encompassing granulocytic and monocytic cells as well as TAM and CD11b^+^ DCs — were less frequent in SI tumors from AOM-treated than untreated mice (24.1 ± 6.1 vs. 53.2 ± 7.0, mean ± SEM, *p* = 0.024; Figure 3A). In contrast, AOM treatment did not seem to alter the frequency of CD11b^+^ cells in colon tumors (Figure 3A). Ly6G^hi^ CD11b^+^ neutrophilic granulocytes were 6-fold more abundant in colon tumors than in SI tumors (AOM treated mice: 59.4 ± 23.2% vs. 19.5 ± 13.9% of CD11b+ cells, *p* < 0.005) (Figure 3B). The percentage of Ly6G^hi^ CD11b^+^ cells was greatly increased in tumors compared to lamina propria of the same AOM-treated *Apc*^1638N/+^ mice in both SI and colon indicating active recruitment of neutrophilic granulocytes to tumors in both locations, but preferential accumulation in colon tumors (Supplementary Figure 2B). Within the CD11b^+^ Ly6G^−^ compartment the proportions of Ly6C^hi^ MHCII^−^ monocytic cells, Ly6C^hi^ MHCII^+^ intermediate cells, Ly6C^−^ MHCII^hi^ CD64^+^ macrophages and Ly6C^−^ MHCII^lo^ CD64^+^ macrophages were similar in colon and SI tumors and not altered by AOM treatment (Figure 3C). The frequency of DCs within CD45^+^ tumor infiltrating cells was not significantly different between the experimental groups and the tumor locations (Figure 3D). CCL2 and CXCL10, showed higher relative mRNA expression levels in tumors than in normal lamina propria of tumor bearing *Apc*^1638N/+^ mice (Figure 3E) correlating with the recruitment of myeloid cells and T lymphocytes into the tumors.

### Distinct Subpopulations of Tumor Infiltrating Myeloid Cells Shape the Intestinal Tumor Microenvironment

**Figure 4:**
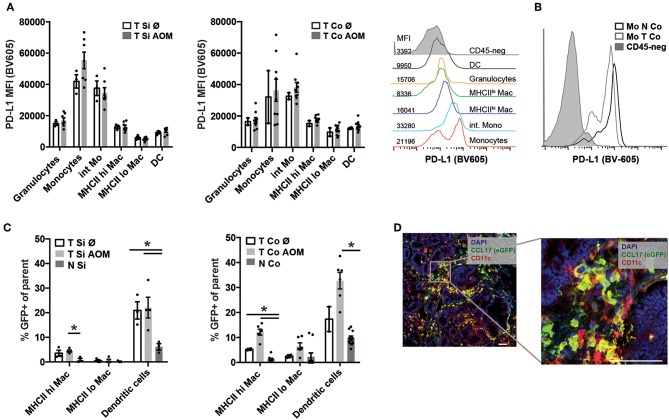
PD-L1 and CCL17 (eGFP) expression in intestinal tumors indicates establishment of a tumor promoting microenvironment. **(A)** PD-L1 mean fluorescence intensity (MFI) of the indicated myeloid cell populations in Si and Co tumors of untreated and AOM treated *Apc*^1638N/+^ mice. Representative histogram overlay of the PD-L1 fluorescence signal in the indicated myeloid cell populations (colored histograms) and CD45-negative tumor cells (gray histogram) from colonic intratumoral cells of an AOM-treated mouse. **(B)** Representative histogram overlay of the PD-L1 fluorescence signal in monocytes from tumor (light gray, open histogram) and normal (dark gray, open histogram) tissue, compared to CD45-negative cells (gray, filled histogram) from colonic intratumoral cells of an AOM-treated mouse. **(C)** CCL17-eGFP expression was detected by flow cytometry in macrophages and DCs in tumors and normal lamina propria of *Apc*^1638N/+^;CCL17-eGFP-reporter mice. The percentages of GFP^+^ cells within the indicated populations are shown. **(A,C)** Each symbol represents an individual mouse. Horizontal bars: mean, error bars: SEM, *n* = 2–9, unpaired two-tailed *t*-test. **(D)** Immunofluorescence staining (left: 20× magnification, scale bar 50 μm) of Si tumor tissue of an AOM-treated mouse. Blue: nuclear staining (DAPI), red: CD11c, green: CCL17-reporter (eGFP). *p < 0.05.

Programmed cell death ligand 1 (PD-L1) interacts with Programmed cell death 1 (PD1) on effector T cells, NK cells and TAMs inhibiting their anti-tumor activity. PD-L1 staining was not detectable on CD45- tumor cells by flow cytometry (Figure 4B) but was found to be expressed on the surface of all myeloid cell subsets within colon and SI tumors irrespective of AOM treatment. Expression levels were highest in Ly6C^hi^ MHCII^−^ followed by Ly6C^hi^ MHCII^+^ monocytic cells and then MHCII^hi^ and MHCII^lo^ TAM subsets (Figure 4A) We detected lower PD-L1 expression on monocytes from the tumors than from lamina propria (Figure 4B) indicating that the tumor microenvironment is less inductive for PD-L1 expression than the lamina propria.

CCL17 expression in the intestine is restricted to DCs in the steady state (37, 38), but is also expressed by immunosuppressive M2 polarized macrophages within tumors (34). Using *Apc*^1638N/+^ CCL17^eGFP/+^ reporter knockin mice (37) CCL17 expression could be detected in TAMs and DCs in tumors of AOM (Figure 4C) at significantly higher levels than in normal lamina propria of tumor bearing mice. Immunofluorescence staining of tumor tissue sections showed infiltrates of CCL17^eGFP^ expressing cells, part of which stained positively for the DC marker CD11c confirming our results from flow cytometric analysis (Figure 4D). Thus, the tumor microenvironment which is established in colon and SI tumors of *Apc*^1638N/+^ mice favors CCL17 expression in TAMs and DCs.

## Discussion

In this study we show that repeated AOM injections increase the incidence and multiplicity of colon tumors in mice with the *Apc*^1638N/+^ germline mutation and shorten experiment time while reducing the small intestinal tumor burden. Tumors developing in AOM treated *Apc*^1638N/+^ mice express WNT target genes and show aberrant accumulation of β-catenin, as expected for WNT driven carcinogenesis. Strikingly, comparison of immune cell infiltrates between colonic and SI tumors revealed a significantly higher frequency of neutrophilic granulocytes in colon tumors. Further, increased expression of CCL17 in DCs and M2-like TAMs within tumors compared to intestinal lamina propria indicates the establishment of a tumor-promoting immunosuppressive environment. Thus, AOM treated *Apc*^1638N/+^ mice can be used as a model of early colon carcinogenesis to further investigate the interplay of immune cells, stromal cells, and cancer cells in the tumor microenvironment.

Repeated AOM administration has been used previously as a model for sporadic CRC. A sufficient incidence of colon tumors can be achieved by AOM administration in the susceptible A/J strain but not in the C57BL/6 strain (39), which is the background of the majority of genetically engineered mouse lines, e.g., for functional studies of relevant immune cell types. Therefore, we sought to combine *Apc*^1638N/+^ mutant mice with AOM treatment and observed greatly increased incidence and multiplicity of colon tumors, demonstrating a synergistic effect of the truncating *Apc*^1638N/+^ mutation and AOM-induced mutations, which led to increased cytoplasmic and nuclear accumulation of β-catenin and upregulation of canonical WNT target genes.

Besides the slightly reduced number of small intestinal tumors, their size was reduced and the majority showed low grade IEN in AOM treated *Apc*^1638N/+^ mice. This is probably due to the earlier termination of the experiment and demonstrates that AOM is specifically affecting the colon. Histologically, high grade IENs and invasive adenocarcinomas were found with comparable frequency in 5–7 months old AOM treated *Apc*^1638N/+^ mice as in 10–12 months old untreated *Apc*^1638N/+^ mice. Thus, AOM treatment accelerated tumor progression, but did not lead to deeper tissue invasion beyond the bowel wall or to metastasis. An increased incidence and number of colon tumors was also reported in *Apc*^min/+^ mice after repeated AOM treatment (23, 24, 26, 40). However, *Apc*^min/+^ mice have a much higher spontaneous tumor burden in the small intestine, and therefore a shorter life time that precludes further progression of lesions along the adenoma-carcinoma sequence. The AOM treated *Apc*^1638N/+^ mice in the present study provide the opportunity to assess the influence of additional risk factors and immune responses on both, lesion incidence and progression to invasive carcinoma. It was shown in a recent study that administration of AOM alone or combined with *Citrobacter rodentium* infection increased proliferation and Dclk1-positive cancer stem cell frequency in intestinal tumors of *Apc*^1638N/+^ mice indicating enhanced tumorigenesis in line with our results (41). However, the impact of AOM administration on tumor multiplicity and progression was not reported in this publication.

Comparison of small intestinal and colon tumors revealed that neutrophilic granulocytes marked by CD11b and Ly6G expression are massively recruited and dominate the myeloid cell infiltrate in colon tumors but not small intestinal tumors. This shows that location has a great impact on the composition of immune cell infiltrates in intestinal tumors, but the regulation of this preferential accumulation of neutrophilic cells in colon tumors is not known. Intratumoral CD11b^+^ Ly6-G^high^ cells consist of classical neutrophils (TANs) and/or pathologically activated immunosuppressive PMN-MDSC, which accumulate in the tumors, but are also found in blood, spleen and bone marrow of tumor bearing mice. TANs are heterogeneous and can have anti-tumor or protumor activity. Their prognostic role in CRC is controversial (42, 43). Although it was shown recently that neutrophils can limit tumor progression in the very early phase of murine CRC models by restricting tumor-associated bacteria and inflammatory responses (44), the majority of reports provide evidence for a tumor supporting role of TANs. PMN-MDSC, which develop at later stages, are by definition immunosuppressive and promote tumor initiation, progression and dissemination (43).

Accumulation of neutrophils/PMN-MDSCs in small intestinal and colon tumors of *Apc*^1638N/+^ mice correlated with increased expression of CCL2 compared to normal intestinal tissue with slightly higher expression in colon tumors. Chun et al. also reported increased CCL2 expression in colitis associated colon cancer, sporadic CRC as well as precancerous colorectal lesions in humans and in mouse models (45). In these models CCL2 was shown to be required for PMN-MDSC and M-MDSC accumulation in the tumors and enhanced the T cell suppressive function of PMN-MDSC (45). In addition to CCL2, CXCR2 ligands including CXCL1, CXCL2, CXCL3, and CXCL8 produced by CRC cells and neutrophils themselves were found to be responsible for recruitment of CXCR2^+^ neutrophils/PMN-MDSCs to colon tumors (46–48). We have shown earlier that specific colon microbiota in human patients are correlated with chemokine production and infiltration of immune cell subsets (49). Preferential recruitment of neutrophilic cells to colon tumors vs. small intestinal tumors could therefore involve site-specific microbiota signals and inflammatory responses (50), but also genetic mechanisms of tumor initiation, which differ between tumor locations in the intestine (7).

Further investigation of tumor infiltrating myeloid cells in intestinal tumors of AOM-treated and untreated *Apc*^1638N/+^ mice confirmed the presence of all subpopulations of monocytes, macrophages and DCs with comparable frequencies in small intestinal and colon tumors. PD-L1 was expressed on tumor-infiltrating monocytic cells, albeit at lower levels than in colon lamina propria. But macrophages, granulocytes and DCs showed only low level expression mimicking the situation in human CRC, where low PD-L1 expression correlates with poor response to PD-1 blockade (51). Successful therapy with pembrolizumab (anti-PD-1) is restricted to the subgroup of mismatch repair deficient CRC which show higher PD-L1 expression (mainly in tumor-associated immune cells) (52). Thus, our model is suitable for investigating regulation of PD-L1 expression in colon tumors and testing therapeutic approaches, which aim to increase responsiveness to checkpoint inhibition.

Using *Apc*^1638N/+^ CCL17-eGFP reporter mice, we found that TAMs and DCs upregulate expression of CCL17 in the microenvironment of small intestinal and colon tumors developing in *Apc*^1638N/+^ mice. CCL17 expression has been used as a marker for M2-like immunosuppressive TAMs and was shown to correlate with Treg frequencies in tumors in line with its ability to attract Tregs expressing CCR4 (34, 53). Therapeutic strategies targeting TAMs (such as CSF-1R inhibitors) were shown to be effective in syngeneic subcutaneous tumor models, but only in combination with immune stimulation or checkpoint blockade (34, 54). Further studies are required to identify novel target molecules, which prevent or revert the immunosuppressive and tumor promoting functions of TAMs and other tumor-infiltrating myeloid cell subpopulations in spontaneously developing cancers, such as the CRC model described here.

The model described here also allows assessing the phenotype of diverse complete and conditional knockout mouse strains with a simple breeding strategy since only one mutated *Apc* allele is necessary, whereas Cre recombinase or complex husbandry regimes are not required for colon tumor formation. This is advantageous compared to models of conditional *Apc* deletion, such as in *Cdx2p*-*Cre*; *Apc*^+/Loxp^ mice (18, 19) or *Fabpl-Cre*; *Apc*^15lox/+^ mice (55) or *Villin-Cre; Tp53* ^Loxp/Loxp^ mice treated with AOM which require backcrossing to the FVB background (56).

We conclude that *Apc*^1638N/+^ mice treated with AOM are a suitable and robust model of colon carcinogenesis and will be useful to develop new strategies for prevention and immunotherapy of CRC.

## Materials and Methods

### Mice

Mice were bred and held in the animal facility of the Institute for Immunology, LMU Munich, Germany under SPF conditions. Health monitoring was performed according to the recommendations of the Federation of European Laboratory Animal Science Association (FELASA). Sentinels occasionally tested positive for *Helicobacter* spp. All experimental procedures involving mice were performed in accordance with the regulations of and were approved by the local government (Regierung von Oberbayern, license no: 55.2-1-54-2532-36-2013). *Apc*^1638N/+^ mice (8) were crossed with CCL17-eGFP reporter mice (37) (all kept on C57BL6/N-background for >10 generations). Starting at the age of 8 weeks mice were injected with 10 mg/kg Azoxymethane (AOM) i.p. weekly for 6 weeks [as described (56)] or were left untreated. Mice were sacrificed by cervical dislocation when reaching criteria for euthanasia, which included clinical signs of anemia.

### Tissue Processing and Single Cell Preparation

The intestines were cut longitudinally, washed with ice-cold PBS and the number, location and size of tumors was recorded. Visible tumors were excised and randomly selected tumors were fixed in 4% PFA for 1 h at 4°C and subsequently incubated in 20% sucrose o/n at 4°C. Tumors were then embedded in OCT (Leica, Wetzlar, Germany) and stored in −80°C. For the generation of single cell suspensions remaining tumor and normal intestinal tissue was cut into 5 mm long pieces and incubated with 2 mM DTT, 10 mM HEPES, 10 mM EDTA for 10 min in a shaking incubator (125 rpm) to dissociate the epithelial layer and then digested with DNAse (0.5 μg/ml), Collagenase D (2.5 μg/ml), Collagenase V (5 μg/ml), and Collagenase IV (157 Wuensch Units/ml) in RPMI-1640 for 30 min at 37°C with gentle shaking before passing through 100 and 70 μm cell strainers.

### Histology

Cryosections (5–8 μm) were incubated in Hematoxylin solution (Merck, Darmstadt, Germany), washed in H_2_O and subsequently stained in Eosin solution (J.T. Baker, Philipsburg, USA), washed in H_2_O, dehydrated and mounted with Roti®-Histokitt (Carl Roth, Karlsruhe, Germany). Histological assessment and grading was performed using a Leica DM2500 after consultation with a pathologist.

### Immunofluorescence Staining

Cyrosections (5–8 μm) were incubated with phosphate buffered saline (PBS) containing 5% goat serum (Vector Labs, Burlingame, USA) and 0.5% Triton-X-100 and then stained with primary antibodies: anti-Ki67 (cat. #12202, Cell signaling technology, Danvers, USA), anti-CD45-FITC (#11-0454-82, Thermo Fisher, Waltham, USA), anti-GFP (#ab6556, Abcam, Cambridge, UK), anti-CD11c (#550283, BD-Bioscience, Franklin Lakes, USA), anti-β-Catenin (#ab22656, Abcam). In case of unlabeled primary antibodies fluorochrome-labeled secondary antibodies were used (#A11008, #A11001, #A21236 Molecular Probes, Eugene, USA). For staining nuclei DAPI (Sigma-Aldrich, St. Louis, USA) was used. Imaging was conducted with a Leica SP8X WLL upright confocal microscope (Leica, Germany).

### Flow Cytometry

Single cell suspensions from normal or tumor tissues, processed as described above, were incubated for 10 min with Fc-blocking reagent (anti-CD16/32 producing hybridoma supernatant) before staining with fluorescently labeled surface antibodies, purchased from Biolegend (CD4, #100547, CD8, #100730, CD19, #115522, MHCII, #107632, CD11b, #101226, Ly6G, #127641, CD64, #139305, CD11c, #117318) and Thermo Fisher (CD3, #47-0031-82, Foxp3, #25-5773-80). For Foxp3-staining the Foxp3-Fix/Perm buffer kit was used according to the manufacturers protocol. The Cytoflex S flow cytometer (Beckman Coulter, Brea, USA) was used and the data were analyzed using FlowJo v. 10 (Tree Star Inc., Ashland, USA).

### RNA Isolation and qRT-PCR

RNA was isolated from OCT-embedded tissue sections using the RNeasy-FFPE Kit (Qiagen, Hilden, Germany) according to the manufacturers protocol. RNA was reverse-transcribed using SuperScript III (Thermo Fisher). For qRT-PCR UPL primer/probe sets for Lgr5, Opn, Ccl2, Cxcl10 (probes: Roche Diagnostics, Rotkreuz, Switzerland; primers: Metabion, Planegg, Germany) or Taqman assays for Hprt1 (Thermo Fisher) were used and qPCR was performed on a LightCycler 480 Real-Time PCR system (Roche). The 2^∧^-ddCT method was used to quantify relative mRNA expression.

### Statistical Analysis

Statistical analysis was performed with GraphPad Prism version 6.0 (GraphPad Software Inc., La Jolla, CA, USA). Normally distributed data was analyzed by unpaired or paired two-tailed *t*-test. Not normally distributed data was analyzed using the Mann-Whitney test. For multiple testing the Holm-Sidak correction method (alpha = 0.05) was used. Survival was analyzed by Kaplan-Meier analysis and log-rank test. The Chi-square test was used to assess relative risk.

## Data Availability Statement

All datasets generated for this study are included in the manuscript/Supplementary Files.

## Ethics Statement

The animal study was reviewed and approved by Regierung von Oberbayern.

## Author Contributions

RM designed and performed experiments and analyzed and interpreted data. SK and MM performed experiments. K-PJ analyzed and interpreted data. AK conceived the project, designed experiments, analyzed and interpreted data, and wrote the manuscript.

### Conflict of Interest

The authors declare that the research was conducted in the absence of any commercial or financial relationships that could be construed as a potential conflict of interest.
