# Supplementary material for: Increased Incidence of Colon Tumors in AOM-Treated Apc1638N/+ Mice Reveals Higher Frequency of Tumor Associated Neutrophils in Colon Than Small Intestine
Source: Front Oncol. 2019 Oct 2;9:1001. doi: 10.3389/fonc.2019.01001 (PMC6797844; doi:10.3389/fonc.2019.01001)

Supplemental information

Increased incidence of colon tumors in AOM-treated *Apc*$^{1638N/+}$ mice reveals higher frequency of tumor associated neutrophils in colon than small intestine

Rebecca Metzger, Mahulena Maruskova, Sabrina Krebs, Klaus-Peter Janssen, Anne B Krug

Inventory of supplemental information:

Supplementary figure 1
Supplementary figure 2

Supplementary figure 1:

(A) Percentages of CD45+ immune cells in tumors of untreated and AOM-treated *Apc*$^{1638N/+}$ mice. (B) Percentages of $CD3^{+}$ T cells, $CD4^{+}$ T-helper cells, $CD4^{+}$ $Foxp3^{+}$ Treg cells and $CD19^{+}$ B cells of all $CD45^{+}$ cells in tumors of untreated and AOM-treated *Apc*$^{1638N/+}$ mice. Each symbol represents an individual mouse. Horizontal bars: mean, error bars: SEM, n=2-9, unpaired two-tailed t-test (not significant).

Supplementary figure 2:

(A) Representative images of CD45 (red) immunofluorescence staining (20x magnification) of small intestinal tumor tissue from untreated (left) and AOM-treated (right) *Apc*$^{1638N/+}$ mice. (B) Percentages of indicated immune cell populations of all $CD45^{+}$ cells in Si and Co tumor lesions (grey, filled bars) and normal intestinal tissue (open bars) of the same individual AOM-treated *Apc*$^{1638N/+}$ mice. Each symbol represents an individual mouse. Lines connect paired values (n=3-6, paired, two-tailed t-test). *$p<0.05$

Supplementary figure 1

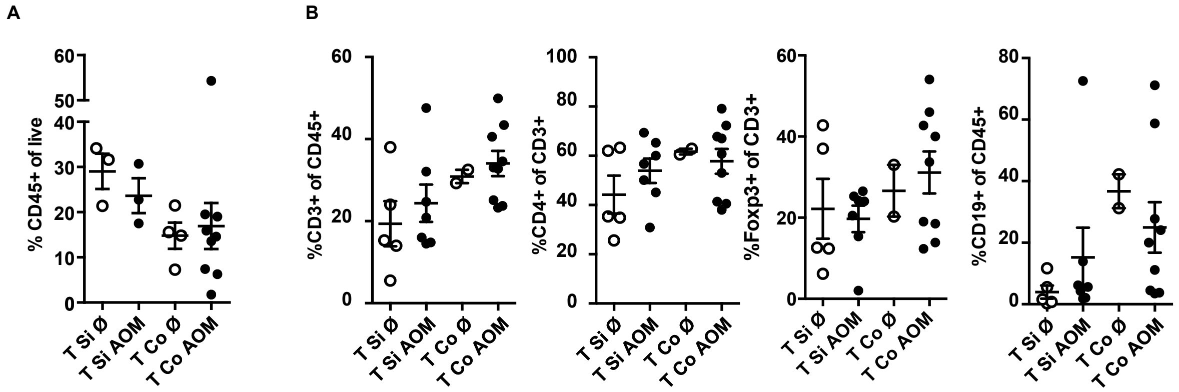

Supplementary figure 2

A

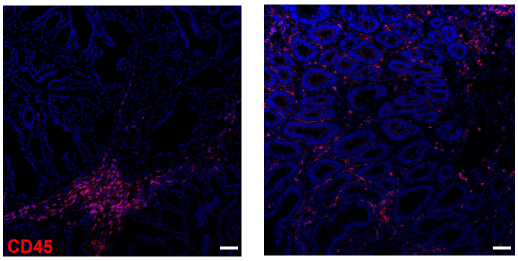

B

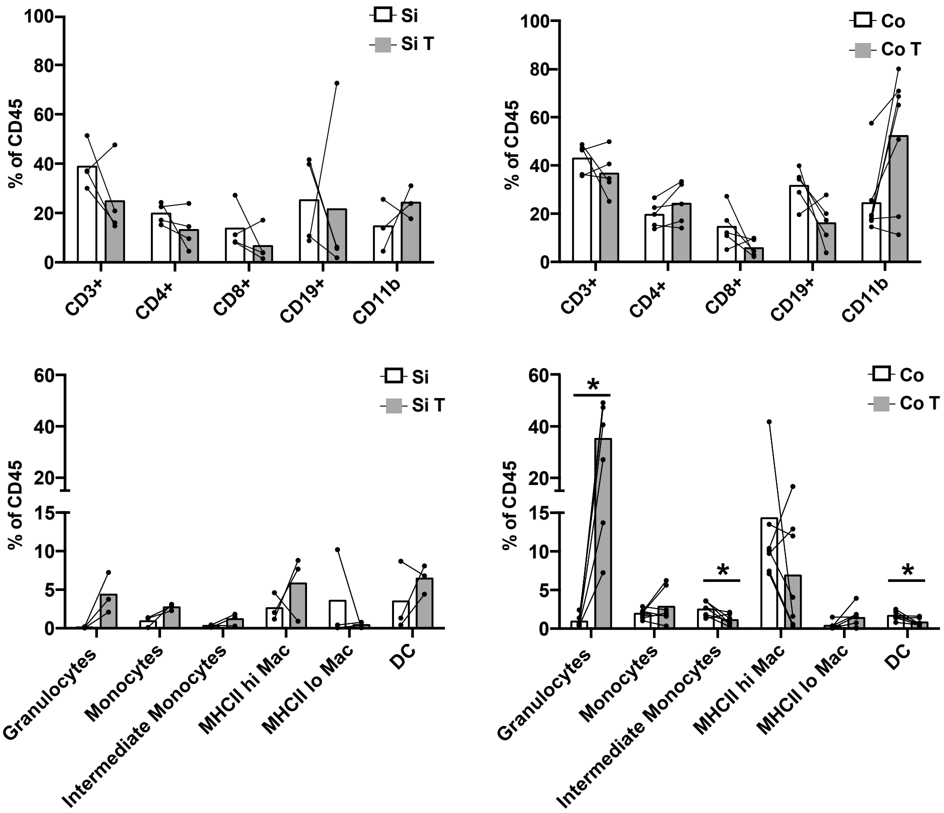

Supplement: Supplementary file 1 [file Data_Sheet_1.PDF]
